# Anti-Inflammatory Effect of an Apigenin-Maillard Reaction Product in Macrophages and Macrophage-Endothelial Cocultures

**DOI:** 10.1155/2019/9026456

**Published:** 2019-05-16

**Authors:** Qian Zhou, Hui Xu, Wenzhe Yu, Edmund Li, Mingfu Wang

**Affiliations:** ^1^School of Biological Sciences, The University of Hong Kong, 518000, Hong Kong; ^2^College of Food Science and Technology, Shanghai Ocean University, Shanghai 200000, China

## Abstract

Chronic inflammation is involved in the progression of various diseases, while dietary flavonoids are reported to possess antioxidative and anti-inflammatory properties against age-related diseases. Previously, an apigenin-Maillard reaction product, dimethylglyoxal apigenin (DMA), was identified by us and demonstrated to be antioxidative. In this study, we investigated the inhibitory effect of DMA on advanced glycation end product- (AGE-) induced inflammation in macrophages and macrophage-endothelial cocultures. Results showed that DMA remarkably inhibited the mRNA and protein expression of receptor for AGEs (RAGE), thereby inhibiting the production of ROS and proinflammatory cytokines, including tumor necrosis factor- (TNF-) *α*, interleukin (IL) 1, IL 6, and monocyte chemoattractant protein- (MCP-) 1 in RAW 264.7 cells. In the coculture system which was performed in the Boyden chamber, macrophage infiltration and adhesion to endothelial cells were significantly suppressed by DMA. Further study indicated that DMA decreased AGE-evoked IL 6 and MCP-1 secretion, which might be achieved through RAGE and its downstream-regulated transforming growth factor- (TGF-) *β*1 and intercellular adhesion molecule (ICAM) 1 expression in the coculture system. In conclusion, our study demonstrates that DMA, a thermally induced compound, has anti-inflammatory activity in both macrophages and macrophage-endothelial cocultures, offering a promising approach for slowing down the development of chronic diseases.

## 1. Introduction

Dietary flavonoids are secondary plant metabolites existing widely in fruits, vegetables, spices, herbs, nuts, and legumes. They are responsible or partially responsible for the colour of flowers and sometimes leaves. Recently, several systematic reviews with meta-analyses of cohort and/or case-control studies have supported the various beneficial effects of flavonoids on chronic diseases such as cardiovascular diseases, type 2 diabetes [1], and some cancers [2, 3]. Flavonoids are very common food ingredients. As an example, quercetin-type flavonoids are of high concentration in onion, hot pepper, and asparagus. However, little attention has been given to the possible chemical reactions that may take place on flavonoids themselves under thermal conditions. Apparently, the structural change of flavonoids will lead to differentiation in bioactivity correspondingly, which also has not been well studied before.

In thermally processing foods, some key food reactions including the Maillard reaction and lipid peroxidation play important roles for food quality. Flavonoids were originally considered to simply act as antioxidants to modulate oxidation processes associating with the above reactions. In recent years, emerging evidence tends to support that certain flavonoids could exhibit probably more critical roles pertinent to their nucleophilic activity. These flavonoids might directly interact with electrophilic intermediary compounds that arise at different stages of the Maillard and/or lipid peroxidation reaction to form novel chemical substances. Lo et al. in 2006 reported the dicarbonyl trapping reaction of green tea catechins under simulated physiological conditions [4]. The reaction products of (-)-epigallocatechin gallate (EGCG) and methylglyoxal (MGO) were separated by a chiral column, and their structures were elucidated by 2D-NMR spectroscopy. This study provided the first line of evidence that certain groups of phenolic antioxidants could directly trap reactive carbonyl species and triggered a reconsideration of the role of flavonoids in the Maillard reaction. More promising data in this area were obtained by D. G. Peterson's group. This research group studied the reactivity of epicatechin (a flavan-3-ol) in the Maillard reaction by using ^13^C, ^15^N-labeled aqueous glucose/glycine model systems [5, 6]. It was found that epicatechin under aqueous conditions could form epicatechin-Maillard reaction products with C_2_, C_3_, and C_4_ sugar fragments.

Some of the newly discovered compounds, formed during heat-induced reactions, have been shown to be potentially antioxidative, anti-inflammatory, and cancer chemopreventive agents. As examples, two ferulic acid-Maillard reaction products (6-(4-hydroxy-3-methoxyphenyl)-5-(hydroxymethyl)-8-oxabicyclo[3.2.1]-oct-3-en-2-one and 2-(6-(furan-2-yl)-7-(4-hydroxy-3-methoxyphenyl)-1-methyl-3-oxo-2,5-diazabicyclo[2.2.2] oct-5-en-2-yl)acetic acid) generated in low-moisture baking model systems suppressed the lipopolysaccharide- (LPS-) mediated expression of two prototypical proinflammatory genes, inducible nitric oxide synthase (iNOS), and cyclooxygenase- (COX-) 2, in an *in vitro* murine macrophage model [7]; a novel quercetin-Maillard reaction product, 8-C-(E-phenylethenyl)quercetin, originally found in onion and beef soup, was identified by our lab and was later proved to prevent colon cancer cell proliferation through inducing autophagic cell death [8]. Evidences of the beneficial effects were also exemplified in studies that propyl gallate-Maillard reaction product (mono-MGO propyl gallate) detected in roasted pork was reported to be antioxidative and against carbonyl stress in lard [9]. However, due to the structural complexity of thermally formed compounds and challenge to obtain enough amounts of thermally formed compounds for bioavailability study, there were very few researches in this area.

Previously, DMA, a Maillard reaction product of apigenin (API, a common dietary flavonoid in celery) and MGO, was firstly identified by us. With the remained hydroxyl group of API but already modified reactive dicarbonyl group of MGO, it is not surprising that DMA retained API's antioxidative properties through the RAGE/ROS/Nrf2 pathway in AGE-induced endothelial damages (under review). However, extensive studies have shown that API has anti-inflammatory capabilities [10]; for instance, API attenuates AGE-induced nitric oxide (NO) generation and TNF-*α* expression in N-11 murine microglia [11]. It is reasonable to hypothesize that DMA might also possess anti-inflammatory abilities, working as a potential therapeutic agent against the progression of chronic diseases. To confirm the hypothesis, we investigate the anti-inflammatory activity of DMA in this study.

Chronic inflammation is linked to the provocation, progression, and exacerbation of many diseases, such as obesity, cancer, and type 2 diabetes [12]. Proverbially, macrophages are the leading contributors to pathological inflammatory processes in response to some danger signals, for instance, LPS and AGEs [13]. Upon the stimulation, macrophages rapidly initiate the production of inflammatory cytokines, including endothelin 1, IL 6, TNF-*α*, and IL-1*β* [14], thus leading to inflammation in the human body. Almost immediately, macrophages and lymphocytes are recruited from the peripheral blood to the endothelial cells, which stands for an early and critical event in the development of vascular dysfunction [15]. In the past, single *in vitro* macrophages were commonly used in published inflammatory research, whereas the coculture model seems to be more effective in representing cell-cell interactions and strengthening the similarities between cell cultures and *in vivo* systems. In coculture, free exchange of substances, such as cytokines, nutrients, and inductive agents, is permitted [16]. Considering that macrophage-endothelial coculture might be a more convincible method for the study of inflammation. Thus, in this study, we evaluate the anti-inflammatory activity of DMA on AGE-induced inflammation using both macrophages and macrophage-endothelial cocultures.

## 2. Materials and Methods

### 2.1. Chemicals and Materials

API was purchased from Xi'an Natural Field Bio-Technique Co. Ltd. (Xi'an, China). DMA was synthesized by incubating API with MGO and MgCl_2_ (1:  20:  4 ratio, MgCl_2_ was used as a catalyst), separated through Sephadex LH-20 open column, and identified by LC/MS and ^1^H NMR analysis. Cell counting kit-8 (CCK-8) was obtained from Dojindo Laboratories (Kumamoto, Japan). Primary antibodies against *β*-actin, RAGE, TGF-*β*1, ICAM 1, and anti-rabbit secondary antibodies conjugated to horseradish peroxidase (HRP) were supplied by Cell Signaling Technology (Boston, MA, USA) and Abcam (Cambridge, MA, USA). Human MCP-1 and IL 6 enzyme-linked immunosorbent (ELISA) kit were offered by Abcam (Cambridge, MA, USA). Other chemicals were purchased from Sigma-Aldrich (St. Louis, MO, USA), if not specifically mentioned.

### 2.2. Cell Culture

RAW 264.7 macrophages were purchased from ATCC (Manassas, VA, USA), which were cultured in high glucose DMEM (Gibco, Life Technologies, Carlsbad, CA, USA) supplemented with 10% fetal bovine serum (FBS; Gibco, Life Technologies, Carlsbad, CA, USA) and 1% Antibiotic-Antimycotic (Gibco, Life Technologies, Carlsbad, CA, USA). Pooled human umbilical vein endothelial cells (HUVECs) were purchased from Lonza Group (Basel, Switzerland). HUVECs were cultured in DMEM/F-12 medium (Gibco, Life Technologies, Carlsbad, CA, USA) supplemented with 20% FBS, 50 mg/L endothelial cell growth supplement (Corning, Bedford, MA, USA), 0.1 g/L heparin sodium salt from porcine intestinal mucosa (Sigma-Aldrich, St. Louis, MO, USA), 1.2 g/L sodium bicarbonate (Sigma-Aldrich, St. Louis, MO, USA), HEPES sodium salt (Sigma-Aldrich, St. Louis, MO, USA), and 1% Antibiotic-Antimycotic. The cells were maintained in a humidified incubator at 37°C containing 5% CO_2_. HUVECs used for all the experiments were of passage 4-9. RAW 264.7 cells were used from passage 3 after reviving from frozen cells.

### 2.3. Cell Viability

Cells (RAW 264.7 macrophages or HUVECs) were seeded into 96-well plates. After adhesion, cells were pretreated with different concentrations of DMA or API for the indicated time intervals. Cell viability was measured with a commercial CCK kit following the manufacturer's instructions [17]. The results were expressed as percentage cell viability.

### 2.4. Preparation of AGEs

The AGEs (methylglyoxal-bovine serum albumin, MGO-BSA) were prepared according to the protocol of Meeprom et al. [18] with slight modifications by incubating 10 mg/mL BSA with 55 mM MGO in 10 mM PBS for 8 days at 37°C. The same procedure, but without MGO, was used to prepare a control BSA. After incubation, both AGEs and control BSA were dialyzed against PBS for 2 days at 4°C. The protein content was determined by the Bradford protein assay using BSA as a reference standard. The level of glycation was measured on a fluorescence spectrophotometer with excitation and emission wavelengths set at 370 and 440 nm, respectively. The relative fluorescence intensity of the AGEs formed was 50-fold compared to the control BSA [19]. The AGEs were stored at -20°C until use.

### 2.5. Measurement of ROS

RAW 264.7 cells were pretreated with 25 *μ*M DMA or API for 24 hrs, followed by stimulation with or without 200 *μ*g/mL of AGEs for 24 hrs. After treatment, cells were detached by trypsin and loaded with 25 *μ*M 2′,7′-dichlorodihydrofluorescein diacetate (DCFH-DA, Sigma-Aldrich, St. Louis, USA) for 1 hr at 37°C in FBS-free medium in the dark. After diffusion into cells, DCFH-DA would be deacetylated followed by oxidation by ROS to form highly fluorescent dichlorofluorescein (DCF), which was determined by flow cytometry (BD FACSAria III, BD Biosciences, USA) [20].

### 2.6. Quantitative Real-Time PCR Analysis (qPCR)

RAW 264.7 cells were pretreated with 25 *μ*M DMA or API for 24 hrs, followed by stimulation with or without 200 *μ*g/mL of AGEs for 24 hrs. After treatment, total RNAs were extracted with the standard TRIzol (Life Technologies, Foster City, CA, USA) method, and the concentrations of RNAs were measured by a spectrophotometer (Nanodrop, Invitrogen, USA). Then, 1 *μ*g of the total RNAs was reverse transcribed using a PrimeScript RT reagent Kit (Takara, Shanghai, China). The mRNA levels of TNF-*α*, IL-1*β*, IL 6, MCP-1, and RAGE were measured by qPCR through LightCycler 96 (Roche, Basel, Switzerland). Values were normalized by 18S expression, and data were analyzed using the *Δ*ΔCT method [21]. The sequences of primers used in qPCR were listed in Table 1.

### 2.7. Western Blot Analysis

After treatment, proteins were extracted with RIPA lysis buffer (Sigma-Aldrich, St. Louis, USA) containing a protease inhibitor cocktail (Sigma-Aldrich, St. Louis, USA). The protein contents were quantified with the BCA Protein Assay Kit. The protein lysate was resolved on 10% SDS-polyacrylamide gel and electrophoretically transferred to polyvinylidene difluoride membranes (PVDF, Bio-Rad). After blocking in 5% nonfat dry milk or BSA in Tris-buffered saline containing 0.1% Tween 20 (TBST) for 1 hr at room temperature, the membranes were incubated with the specific primary antibodies at 4°C overnight. The membranes were then washed and incubated with the appropriate secondary antibodies for 1 hr. Protein bands were visualized with a chemiluminescence detection kit (ECL, Thermo Fisher Scientific). Quantification was performed using ImageJ software (National Institute of Health, Bethesda, MD, USA). *β*-Actin was served as the loading control [22].

### 2.8. Macrophage Infiltration and Adhesion in the Coculture System

The Boyden chamber assay was used to evaluate the migration of macrophages to endothelial cells [23]. HUVECs were seeded on coverslips in a 24-well plate which were coated by 0.01 mg/mL poly-L-lysine solution (MW 150,000-300,000, Sigma) and cotreated by AGEs (500 *μ*g/mL), DMA (10 *μ*M) + AGEs (500 *μ*g/mL), or API (10 *μ*M) + AGEs (500 *μ*g/mL) for 24 hrs. Then, the Transwell inserts (5.0 *μ*m pore size polycarbonate membrane, Corning Costar Corporation, Cambridge, MA) were placed into a 24-well plate; macrophages were seeded in the inserts (the upper compartment) of the Transwell and cocultured with HUVECs for further 24 hrs. Then, cells in the upper compartment were used for the measurement of infiltration, while the lower compartment for macrophage adhesion. The whole microporous membranes (the upper compartment) and the coverslips (the lower compartment) were fixed with methanol for 20 mins, stained with 0.1% crystal violet for 10 mins, and washed with PBS. Then, the upper side of the membranes was carefully wiped with cotton to remove cells that did not pass through the membrane, followed by clipping with scissors (for microphage observation) or washed off by 30% acetic acid (for macrophage counting determination). The coverslips were ready for morphology observation or counting determination (washed off by 30% acetic acid). For cell counting determination, the solution was transferred into a 96-well plate and measured at the wavelength of 580 nm [15].

### 2.9. ELISA Assay

The supernatants from the coculture system were collected for the determination of the levels of IL 6 and MCP-1 using an ELISA kit according to the manufacturer's recommendations. Quantification was performed on a microplate reader set at 450 nm [24].

### 2.10. Statistical Analysis

All experiments were repeated at least three times. The data were analyzed using one-way analysis of variance (ANOVA) followed by Turkey's test for comparisons of group means. All statistical analyses were performed using SPSS for Windows, version 22 (SPSS, Inc.). *p* values of <0.05 are considered statistically significant. Graphs were drawn by GraphPad Prism version 6.0 for Windows (GraphPad Software Inc.).

## 3. Results and Discussion

### 3.1. DMA Decreased AGE-Induced Oxidative Stress in Macrophages

Evidences indicate that oxidative stress, which is defined as excessive production of ROS, can lead to cellular redox imbalance and plays a pathogenic role in many chronic diseases [25]. Therefore, the reduction of oxidative stress is a strategy for delaying the occurrence of chronic diseases. It has been well documented that AGEs induce ROS overproduction in a quantity of cell types, including macrophages, endothelial cells, and chondrocytes [21].

**Figure 1:**
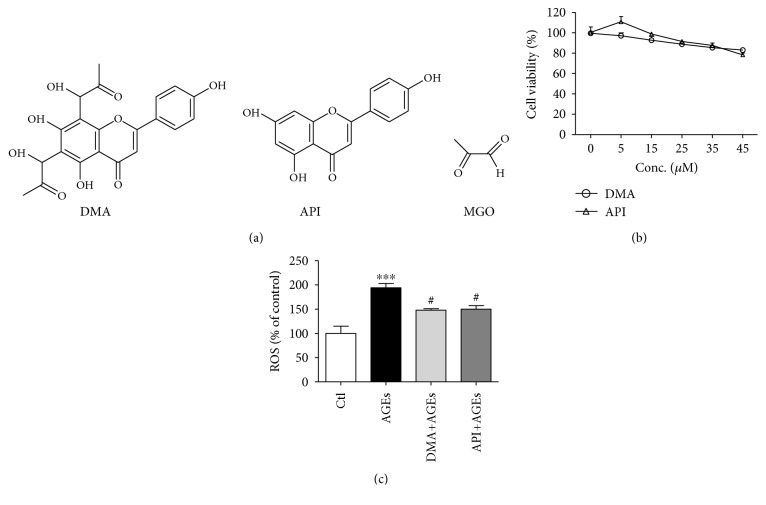
DMA suppresses AGE-induced oxidative stress in macrophages. (a) Chemical structures. (b) Cell viability of DMA and API assayed with CCK kit. The incubation time for DMA and API was 24 hrs. (c) ROS intensity by flow cytometry. RAW 264.7 macrophages were pretreated with 25 *μ*M DMA or API for 24 hrs before stimulation with/without 200 *μ*g/mL of AGEs for 24 hrs. ^∗∗∗^ *p* < 0.001 vs. control; ^#^ *p* < 0.05 vs. AGE treatment.

The structure of DMA, API, and MGO was demonstrated in Figure 1(a), from which we could see that the basic skeleton of API was remained for DMA while two additional side chains were introduced with the addition of two molecules of MGO. The parental compound API was adopted as a control in the following experiments. To figure out a proper dosage in RAW 264.7 cells, cell viabilities were measured by the CCK assay. As shown in Figure 1(b), the cell viability decreased correspondingly when incubated with increased DMA or API (0-45 *μ*M) for 24 hrs. Eventually, 25 *μ*M of DMA and API which showed almost no influence on cell growth was adopted. In addition, 200 *μ*g/mL of AGEs was chosen based on our previous work (cell viability around 90%). To investigate the effect of DMA against AGE-induced ROS production, RAW 264.7 macrophages were stimulated with 200 *μ*g/mL of AGEs for 24 hrs with/without DMA and API (25 *μ*M, 24 hrs) pretreatment. Results (Figure 1(c)) showed that the pretreatment of DMA significantly (*p* < 0.05) lowered ROS level compared with that in only AGE-treated macrophages, which demonstrated the antioxidative capability of DMA under the tested conditions. However, the effectiveness of DMA in comparison with API showed no significance. API and isovitexin (apigenin-6-*C*-glucoside) have been reported to scavenge and stabilize free radicals, thereby reducing oxidative damages in the past [26, 27]. With the same aglycone, it might be inferred that the antioxidant activity of DMA was through scavenging excessively induced ROS.

### 3.2. DMA Counteracted AGE-Induced Inflammation in Macrophages

**Figure 2:**
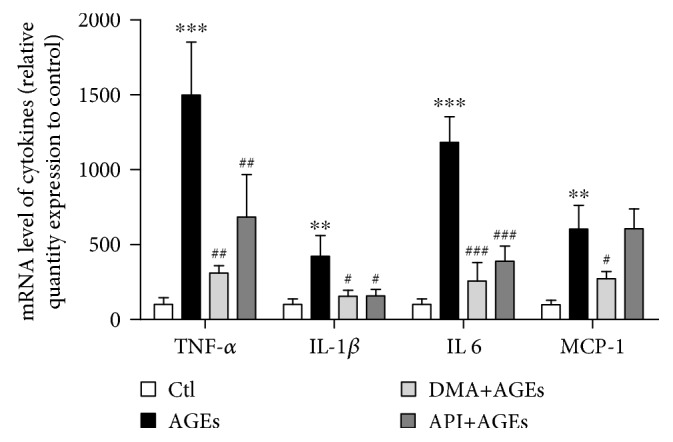
DMA ameliorates AGE-induced inflammatory cytokine expression in macrophages. Relative mRNA levels by qPCR. RAW 264.7 macrophages were pretreated with 25 *μ*M DMA or API for 24 hrs before stimulated with/without 200 *μ*g/mL of AGEs for 24 hrs. ^∗∗^ *p* < 0.01 vs. control, ^∗∗∗^ *p* < 0.001 vs. control; ^#^ *p* < 0.05 vs. AGE treatment, ^##^ *p* < 0.01 vs. AGE treatment, ^###^ *p* < 0.001 vs. AGE treatment.

The increased expression of proinflammatory cytokines in macrophages by the stimulation with AGEs has been previously demonstrated both *in vitro* and *in vivo* [28, 29]. These proinflammatory cytokines, including TNF-*α*, IL-1*β*, IL 6, and MCP-1, would promote systemic inflammation which contributes to the progression of vascular dysfunction, diabetic vasculopathy, and other diseases [15]. To evaluate the effect of DMA on AGE-induced inflammatory responses, qPCR was performed to detect the transcriptional changes of TNF-*α*, IL-1*β*, IL 6, and MCP-1 in RAW 264.7 cells. In accordance with expectations, AGEs (200 *μ*g/mL, 24 hrs) significantly (*p* < 0.01 - *p* < 0.001) increased the mRNA levels of these proinflammatory cytokines in RAW 264.7 macrophages, while the pretreatment of macrophages with 25 *μ*M DMA for 24 hrs notably decreased the levels of TNF-*α*, IL-1*β*, IL 6, and MCP-1 by ~79% (*p* < 0.01), 63% (*p* < 0.05), 78% (*p* < 0.01), and 55% (*p* < 0.05), respectively (Figure 2). Our results indicated that DMA was remarkably effective in mediating AGE-induced inflammation in RAW 264.7 cells. API had an established reputation against inflammation [30] and was also proved to inhibit AGE-evoked TNF-*α*, IL-1*β*, and IL 6 gene expression in the present study. Intriguingly, the anti-inflammatory effectiveness of DMA was significantly stronger than API in terms of MCP-1 and was higher (although no significance) in terms of TNF-*α*. Choi et al. [31] reported that *C*-glycosylation at C6/C8 position of the A ring decreased the anti-inflammatory potential of luteolin (structural similitude with API except an extra hydroxyl group at C3′ position of the B ring) in LPS-stimulated RAW 264.7 cells, whereas our data implied that MGO binding to the C6 and C8 position might, at least partly, strengthen API's anti-inflammatory effects. Besides, evidences also exemplified that API derivatives, apigenin-7-*O-β*-D-glucuronide isolated from the fruit husks of *Juglans sigillata*, inhibited the LPS-induced expression of proinflammatory cytokines in the same cell line [32] and schaftoside (6-*C-β*-glucopyranosyl-8-*C-α*-arabinopyranosylapigenin) identified from the aerial parts of *Eleusine indica* was able to prevent airway inflammatory process *in vivo* [33]. More researches are needed to further figure out the structure-activity relationship. However, this is the first time to report that an apigenin-type compound is effective against AGE-induced inflammation.

### 3.3. DMA Decreased AGE-Induced Inflammation via RAGE in Macrophages

**Figure 3:**
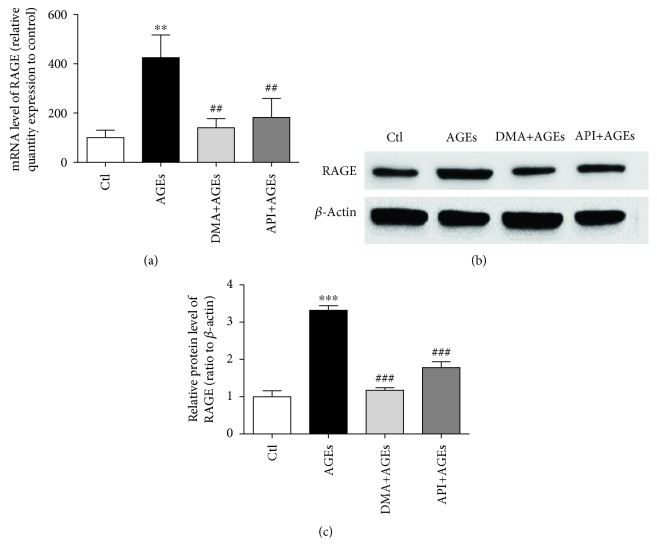
DMA ameliorates the AGE-induced RAGE expression in macrophages. RAW 264.7 macrophages were pretreated with 25 *μ*M DMA or API for 24 hrs before stimulation with/without 200 *μ*g/mL of AGEs for 24 hrs. (a) RAGE mRNA expression by qPCR. (b) RAGE protein expression by Western blot. Total cell lysates were obtained by lysis in RIPA buffer containing protease inhibitors. *β*-Actin served as a control. (c) RAGE protein fold changes were quantified with ImageJ software. ^∗∗^ *p* < 0.01 vs. control, ^∗∗∗^ *p* < 0.001 vs. control; ^##^ *p* < 0.01 vs. AGE treatment, ^###^ *p* < 0.001 vs. AGE treatment.

RAGE, a member of the immunoglobulin superfamily of cell surface molecules, is involved in intracellular signaling resulting in the rapid activation of proinflammatory master regulator NF-*κ*B; simultaneously, the activation of RAGE downregulates cellular defence mechanisms leading to the loss of intrinsic antioxidant activities, including reduced glutathione (GSH), superoxide dismutase (SOD), and catalase (CAT) [34]. It is repeatedly demonstrated that AGEs can interact with RAGE, increase the expression of RAGE, and amplify the cascade of a series of reactions [35]. Our data showed that the RAGE gene level was increased to 425% by AGE treatment (200 *μ*g/mL, 24 hrs), whereas DMA (25 *μ*M, 24 hrs) significantly decreased the AGE-induced gene expression to 140% compared with the untreated cells (Figure 3(a)). The change of the RAGE protein level further verified the beneficial effects of DMA on the AGE-induced RAGE expression. As shown in Figures 3(b) and 3(c), the treatment of macrophages with 200 *μ*g/mL of AGEs for 24 hrs elevated the protein level of RAGE to ~1.2-fold. The pretreatment of cells with 25 *μ*M DMA for 24 hrs significantly moderated the stimulating effect of AGEs (*p* < 0.001), bringing the protein expression of RAGE back to the unstimulated level. API (25 *μ*M, 24 hrs) also ameliorated the AGE-induced gene and protein expression of RAGE, but the inhibitory effectiveness of the protein level was significantly lower than DMA. Zhang et al. [36] demonstrated that liquiritin (a liquiritigenin-type flavonoid) alleviated AGE-induced inflammation in HUVECs via blocking the RAGE/NF-*κ*B/inflammation negative feedback pathway, while Chandler et al. [11] showed that API prevented LPS-evoked NF-*κ*B activation in mouse macrophages. Thus, it might be concluded that the action mechanism of DMA against AGE-induced inflammation is also through the AGE/RAGE/NF-*κ*B signaling pathway.

### 3.4. DMA Prevented AGE-Induced Macrophage Infiltration and Adhesion to Endothelial Cells

**Figure 4:**
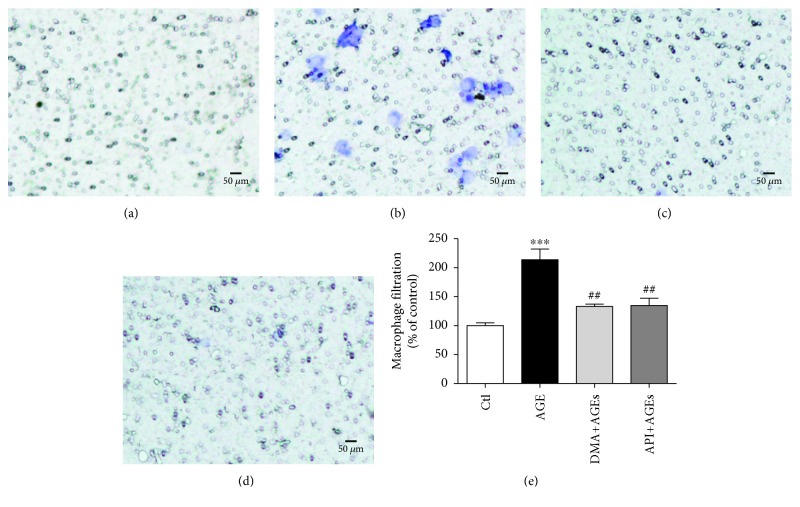
DMA protects AGE-induced macrophage infiltration. RAW 264.7 macrophages at the upper compartment of the Boyden chamber were incubated with medium (a), 500 *μ*g/mL AGEs (b), 10 *μ*M DMA + 500 *μ*g/mL AGEs (c), 10 *μ*M API + 500 *μ*g/mL AGEs (d) for 24 hrs. Filtered macrophages in the microporous membrane were stained by 0.1% crystal violet, and representative photos were taken under a microscope. (e) Macrophage counting. The stain was washed off by 30% acetic acid and measured at a wavelength of 580 nm. ^∗∗∗^ *p* < 0.001 vs. control; ^##^ *p* < 0.01 vs. AGE treatment.

**Figure 5:**
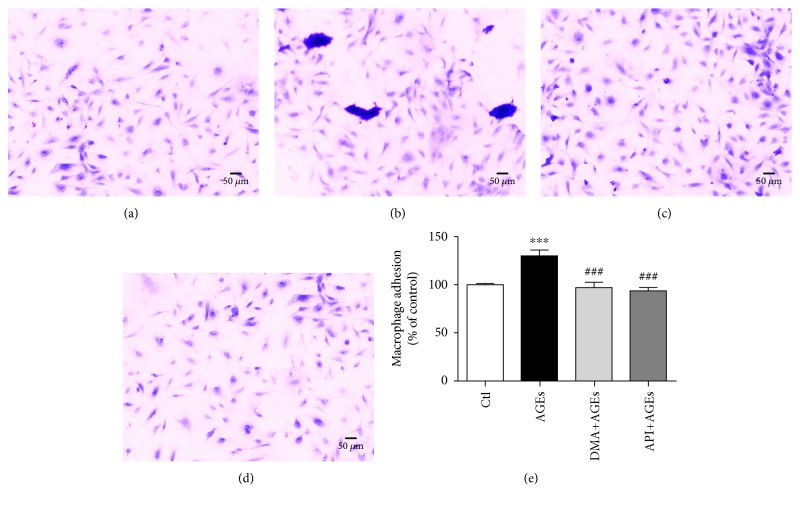
DMA prevents AGE-induced macrophage adhesion to endothelial cells. HUVECs at the lower compartment of the Boyden chamber were incubated with medium (a), 500 *μ*g/mL AGEs (b), 10 *μ*M DMA + 500 *μ*g/mL AGEs (c), or 10 *μ*M API + 500 *μ*g/mL AGEs (d) for 48 hrs. Macrophages adhered to HUVECs were stained by 0.1% crystal violet, and representative photos were taken under a microscope. (e) Macrophage counting. The stain was washed off by 30% acetic acid and measured at a wavelength of 580 nm. ^∗∗∗^ *p* < 0.001 vs. control; ^###^ *p* < 0.001 vs. AGE treatment.

The inflammatory cell infiltration within vasculature was engaged in the development of many diseases, for instance, as an early indicator/change in experimental diabetes [37]. In the present study, we have already observed that AGEs evoked inflammation in RAW 264.7 cells, whereas DMA significantly decreased the inflammatory responses. To further clarify whether DMA could defend against AGE-induced infiltration and adhesion of inflammatory cells to endothelial cells, RAW 264.7 macrophage-HUVEC coculture and the Boyden chamber assay were used. According to our previous work, the concentration of 500 *μ*g/mL for AGEs and 10 *μ*M for DMA and API was employed in the cocultures as the cell viability of HUVECs will not be significantly affected at the specific level. The results showed that AGE treatment (500 *μ*g/mL) increased macrophage infiltration by ~1.1-fold in the upper compartment, whereas cotreatment with DMA (10 *μ*M) remarkably (*p* < 0.01) abrogated this enhancement (Figure 4). The adhesion of macrophages to HUVECs, which was detected from the lower compartment, was also significantly increased by ~30% when comparing AGE treatment with the control, and the cotreatment with DMA led the inflammatory cell adhesion to the normal state (Figure 5). API was also effective in preventing AGE-induced macrophage infiltration and adhesion to HUVECs and showed no significant difference with DMA (Figures 4-5). This is the first time to report the protective effect of API and its derivative against AGE-induced macrophage infiltration and adhesion in macrophage-endothelial cocultures.

### 3.5. Inhibitory Effect of DMA on AGE-Induced Inflammation in Macrophage-Endothelial Cocultures

**Figure 6:**
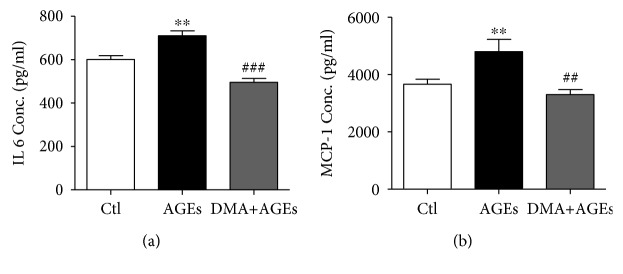
DMA ameliorates AGE-induced inflammatory cytokines in macrophage-endothelial cocultures. (a) Protein secretion of IL 6. (b) Protein secretion of MCP-1. After treatments, supernatants in the Boyden chamber were collected and assayed by ELISA kits. The incubation time with AGEs was 48 hrs. ^∗∗^ *p* < 0.01 vs. control; ^##^ *p* < 0.01 vs. AGE treatment, ^###^ *p* < 0.001 vs. AGE treatment.

As inflammatory biomarkers, IL 6 and MCP-1 are believed to play pivotal roles in AGE-induced inflammation [38]. IL 6, with a wide range of biological activities in immunoregulation, once dysregulated, might lead to systemic inflammatory manifestation in patients with arthritis [39]. MCP-1 is critical in chronic inflammatory diseases by initiating monocyte recruitment to the vessel walls, and the MCP-1 level in blood circulation is associated with dietary AGE consumption [40]. To evaluate the effect of DMA on AGE-induced inflammation in RAW 264.7 and HUVECs coculture system, the secretions of IL 6 and MCP-1 were measured by ELISA kits. Our data showed that AGEs (500 *μ*g/mL) significantly increased the IL 6 and MCP-1 expression in the culture supernatant (*p* < 0.01, vs. nontreated cells) as depicted in Figure 6, while DMA (10 *μ*M) and AGE (500 *μ*g/mL) cotreatment dramatically decreased IL 6 (*p* < 0.001) and MCP-1 (*p* < 0.01) level as compared with AGE-treated groups.

### 3.6. DMA Decreased AGE-Induced Inflammation through RAGE in Macrophage-Endothelial Cocultures

**Figure 7:**
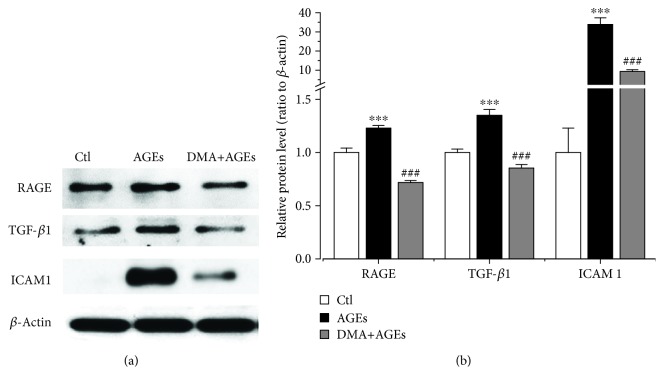
DMA decreases AGE-induced inflammation through RAGE in macrophage-endothelial cocultures. (a) Protein expression by Western blot. HUVECs at the lower compartment of the Boyden chamber were incubated with medium, 500 *μ*g/mL AGEs, or 10 *μ*M DMA + 500 *μ*g/mL AGEs for 48 hrs. Total cell lysates were obtained by lysis in RIPA buffer containing protease inhibitors. *β*-Actin served as a control. (b) Fold changes were quantified with ImageJ software. ^∗∗∗^ *p* < 0.001 vs. control; ^###^ *p* < 0.001 vs. AGE treatment.

Previously, in an *in vitro* atherosclerosis model which was performed with monocyte-HUVEC-VSMC (vascular smooth muscle cell) cocultures, RAGE was reported to regulate AGE-induced endothelium dysfunction through the ERK/NF-*κ*B pathway. However, RAGE was only expressed in monocytes and HUVECs in the particular cocultures and promoted the proliferation of VSMCs afterwards [41]. The macrophage-endothelial coculture model (RAW 264.7 cells and HUVECs) was also used in AGE-modulated inflammation, and a natural isoflavone, calycosin, and its glycoside calycosin-7-*O-β*-D-glucopyranoside were demonstrated to mediate AGE-induced RAGE behaviour [37, 42]. In this study, we further evaluated the protective effect of DMA against the overexpression of RAGE in HUVECs using the same macrophage-endothelial cocultures. Results showed that DMA (10 *μ*M) + AGE (500 *μ*g/mL) treatment in the coculture model dramatically reduced the protein level of RAGE, which was even ~30% lower than the untreated cells (Figure 7). However, DMA treatment offered similar outcome with the unstimulated control in the single macrophage model (Figures 3(b) and 3(c)), suggesting that DMA might be more effective in the *in vivo*-like environment.

It has been well documented that TGF-*β*1 plays a key role in the progression of diabetes and its complications partly by modulating inflammation through the upregulation of NF-*κ*B, MCP-1, and IL 1 expression [43]. It was also reported that TGF-*β*1, which promoted the synthesis of matrix proteins within the basement membrane of endothelial cells at pathological status, participated in AGE-induced macrophage filtration and adhesion to endothelial cells, thus exacerbating the process of inflammation [37]. Chemical agent which targets TGF-*β*1 would be of beneficial effect. As an example, T5Z-7-oxozeaenol, an inhibitor of TGF-*β*-activated kinase 1 (TAK1), could prohibit AGE-induced macrophage activation and decrease inflammatory cytokine levels through the RAGE/MAPK/NF-*κ*B pathway [44]. To further evaluate DMA's effect on AGE-induced macrophage migration, the TGF-*β*1 protein expression in HUVECs was measured by adopting the coculture system. Our results showed that DMA (10 *μ*M) remarkably (*p* < 0.001) decreased the AGE-induced (500 *μ*g/mL) TGF-*β*1 level (Figure 7) to even lower than the unstimulated control. As a member of the immunoglobulin superfamily, ICAM 1 is expressed on the membranes of many cells, including epithelial cells, leukocytes, and endothelial cells [45]. At an early stage of diabetic nephropathy and retinopathy, the influx of inflammatory cells (e.g., macrophages) to endothelial cells is mainly modulated by adhesion molecules, such as ICAM 1 [40]. The role of ICAM 1 in inflammatory responses is well documented. In addition, it has been observed that TGF-*β*1 induced neutrophil-mediated lung injury via upregulation of ICAM 1 in endothelial cells [46]. To better understand the protective mechanism of DMA on AGE-induced inflammation, the protein level of ICAM 1 expressed by HUVECs in the macrophage-endothelial cocultures in response to treatment with AGEs was measured. As expected, AGEs (500 *μ*g/mL) alone dramatically (*p* < 0.001) increased their expression by 330% compared with the unstimulated controls, while DMA (10 *μ*M) + AGEs (500 *μ*g/mL) effectively attenuated the AGE-induced overexpression of ICAM 1 by 72% compared with only AGE treatment (Figure 7). Our data indicated that the AGE-RAGE axis increased the TGF-*β*1 level, which might subsequently result in the expression of ICAM 1, and then ICAM 1 modulated the infiltration and adhesion of macrophages to endothelial cells, amplifying the inflammatory response. Anyhow, DMA could inhibit RAGE expression thus reducing AGE-RAGE interaction and preventing the expression of TGF-*β*1 and ICAM 1, eventually inhibit the cascade of inflammation.

## 4. Conclusions

**Figure 8:**
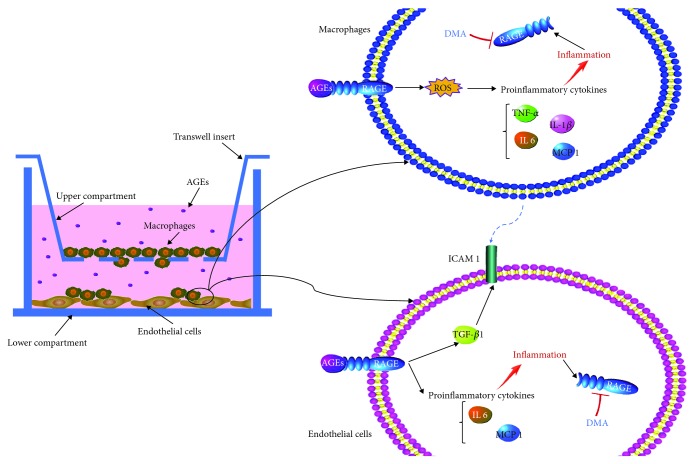
Schematic diagram of DMA on AGE-induced inflammation in macrophages and macrophage-endothelial cocultures.

In this study, the anti-inflammatory abilities of DMA were demonstrated in macrophages and macrophage-endothelial cocultures. As shown in Figure 8, the AGE-RAGE axis on the one hand evoked the overexpression of ROS and proinflammatory cytokines, including TNF-*α*, IL 1, IL 6, and MCP-1, leading to inflammatory status in macrophages. Inflammation in return increased the expression of RAGE and thus promoted the interaction of AGEs and RAGE. On the other hand, the AGE-RAGE axis induced inflammation in endothelial cells and upregulated the level of ICAM 1 through TGF-*β*1, whereas the stimulus of overinduced ICAM 1 impelled the macrophages to endothelial cells, thus exacerbating the process of inflammation and accelerating the development of vascular dysfunction. DMA (an apigenin-Maillard reaction product) could prevent AGE-induced inflammation via ameliorating the expression of RAGE in both macrophages and endothelial cells. Therefore, DMA might be a therapeutic agent for chronic inflammatory disorders, which also offers a hint to consider and further evaluate the health benefits of other flavonoid-Maillard reaction products formed during thermal processing.

## Figures and Tables

**Table 1:** Primers used for qPCR analysis.

Gene	Forward primer	Reverse primer
TNF-*α*	CACCACGCTCTTCTGTCTACTG	CTTTGAGATCCATCGCGTTG
IL-1*β*	GCAACTGTTCCTGAACTCAACT	ATCTTTTGGGGTCCGTCAACT
MCP-1	AGCTCTTTCCTCCACCA	CTACAGCTTCTTTGGGACACCT
IL 6	AGCCAGAGTCCTTCAGAGAGAT	GCACTAGGTTTGCCGAGTAGAT
RAGE	AACACAGCCCCCATCCAA	GCTCAACCAACAGCTGAATGC
18S	GTAACCCGTTGAACCCCATT	CCATCCAATCGGTAGTAGCG

## Data Availability

The data used to support the findings of this study are included within the article.

## Supplementary Materials

Supplementary Materials Figure 1: the total ion chromatogram (TIC) and extracted ion chromatogram (XIC) of DMA detected by HPLC-Q-TOF-MS. (a) TIC. (b) XIC. Positive mode was adopted, collision energy = 10 V, ionspray voltage at 5500 V, ion scan from 50 to 600 Da, and temperature at 400°C. [M + H] + at *m*/*z* 415.0983, *m*/*z* 397.0889 means a molecule depletion of H_2_O, and *m*/*z* 379.0786 means two molecule depletion of H_2_O. Figure 2: 1H NMR of DMA. A 400 MHz Bruker instrument (Billerica, MA, USA) was adopted at 25°C, with compound dissolved in CD_3_OD in a 3 mm i.d. tube. The chemical shift assignments were referenced internally to TMS at 0 ppm.
