# Supplementary material for: Anti-Inflammatory Effect of an Apigenin-Maillard Reaction Product in Macrophages and Macrophage-Endothelial Cocultures
Source: Oxid Med Cell Longev. 2019 May 16;2019:9026456. doi: 10.1155/2019/9026456 (PMC6541947; doi:10.1155/2019/9026456)
Supplement: Supplementary Materials — Figure 1: the total ion chromatogram (TIC) and extracted ion chromatogram (XIC) of DMA detected by HPLC-Q-TOF-MS. (a) TIC. (b) XIC. Positive mode was adopted, collision energy = 10 V, ionspray voltage at 5500 V, ion scan from 50 to 600 Da, and temperature at 400°C. [M + H] + at m/z 415.0983, m/z 397.0889 means a molecule depletion of H2O, and m/z 379.0786 means two molecule depletion of H2O. Figure 2: 1H NMR of DMA. A 400 MHz Bruker instrument (Billerica, MA, USA) was adopted at 25°C, with compound dissolved in CD3OD in a 3 mm i.d. tube. The chemical shift assignments were referenced internally to TMS at 0 ppm. [file 9026456.f1.pdf]

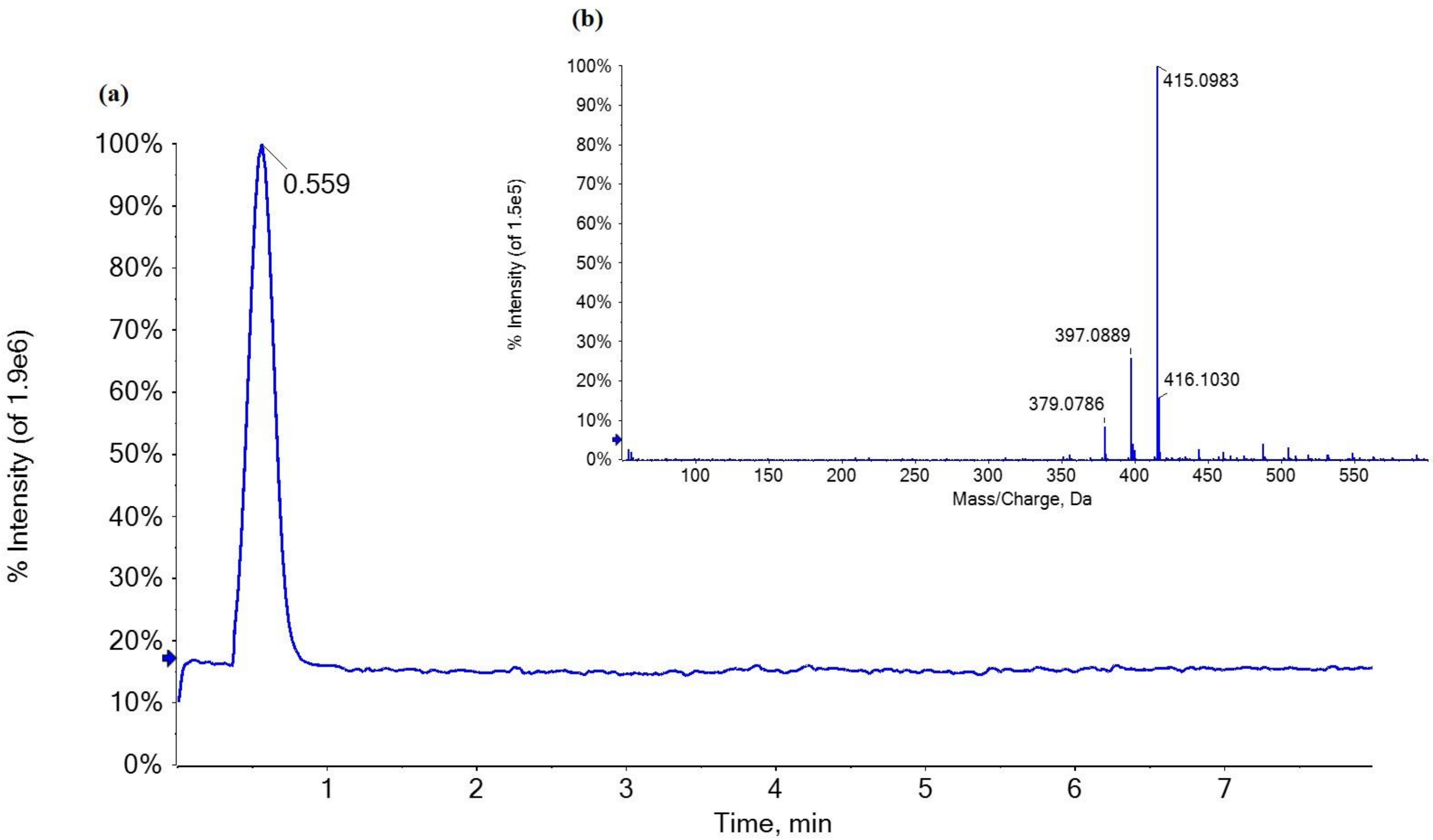

Figure 1: The total ion chromatogram (TIC) and extracted ion chromatogram (XIC) of DMA detected by HPLC-Q-Tof-MS. (a) TIC. (b) XIC. Positive mode was adopted, collision energy=10 V, ionspray voltage at 5500 V, ion scan from 50-600 Da, temperature at 400 °C. $[M+H]^+$ at m/z 415.0983, m/z 397.0889 means a molecule depletion of H_2O, m/z 379.0786 means two molecule depletion of H_2O.

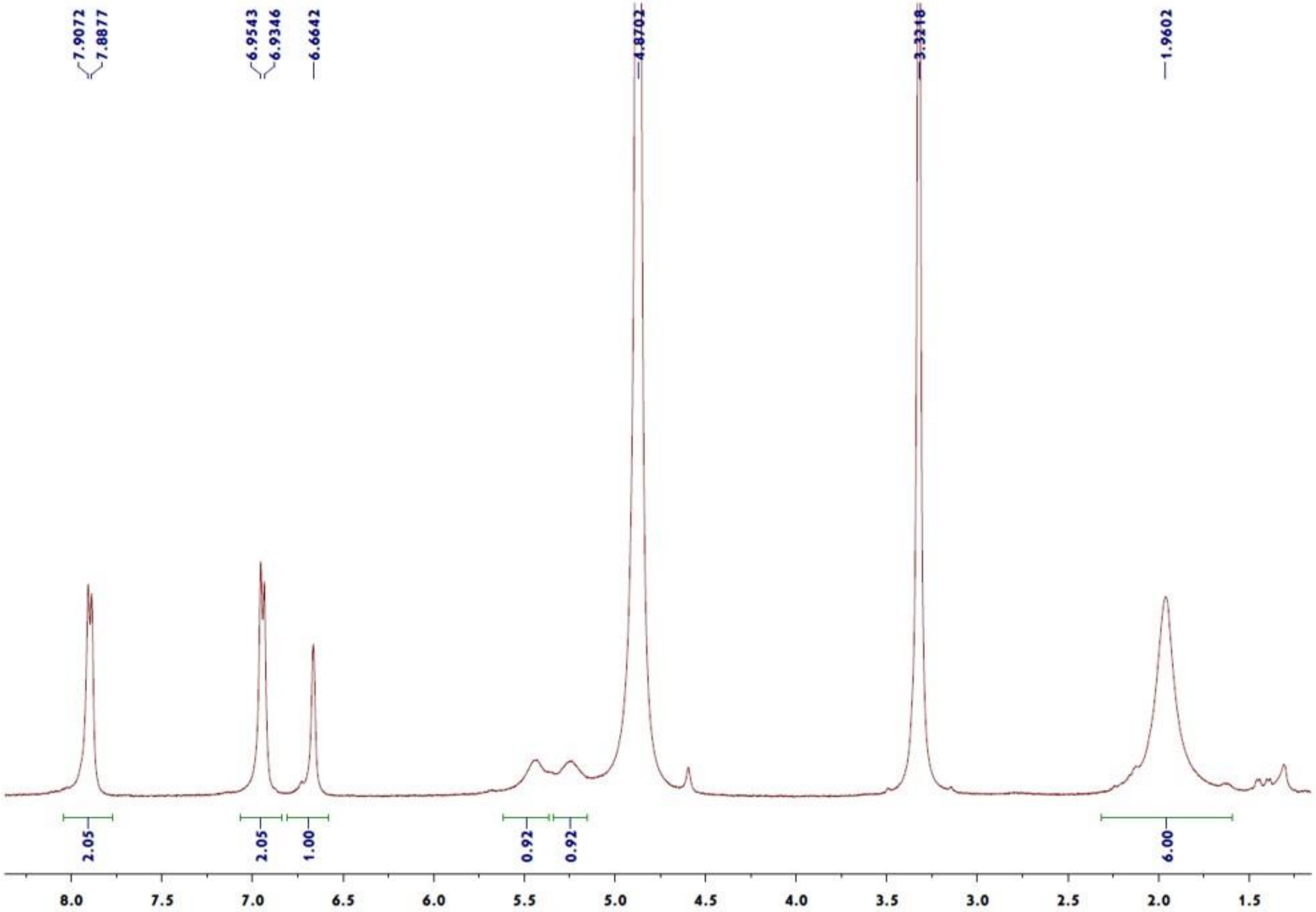

Figure 2: ^{1}H NMR of DMA. A 400 MHz Bruker instrument (Billerica, MA, USA) was adopted at 25 °C, with compound dissolved in CD_3OD in a 3 mm i.d. tube. The chemical shift assignments were referenced internally to TMS at 0 ppm.
